# Muscular dystrophy patients show low exercise‐induced blood flow in muscles with normal strength

**DOI:** 10.1002/acn3.52194

**Published:** 2024-09-09

**Authors:** Orna Gera, Efrat Shavit‐Stein, Taly Amichai, Joab Chapman, Odelia Chorin, Lior Greenbaum, Amir Dori

**Affiliations:** ^1^ Department of Neurology Sheba Medical Center Tel Hashomer Ramat Gan Israel; ^2^ Department of Physical Therapy, Faculty of Medicine Tel Aviv University Tel Aviv Israel; ^3^ Department of Neurology and Neurosurgery, Faculty of Medicine Tel‐Aviv University Tel‐Aviv Israel; ^4^ Robert and Martha Harden Chair in Mental and Neurological Diseases, Faculty of Medicine Tel Aviv University Tel Aviv Israel; ^5^ Faculty of Medicine Tel‐Aviv University Tel‐Aviv Israel; ^6^ The Danek Gertner Institute of Human Genetics, Sheba Medical Center Tel Hashomer Israel

## Abstract

**Objective:**

Neuromuscular evaluation increasingly employs muscle ultrasonography to determine muscle thickness, mean grayscale echointensity, and visual semiquantitative echotexture attenuation. However, these measures provide low sensitivity for detection of mild muscle abnormality. Exercise‐induced intramuscular blood flow is a physiologic phenomenon, which may be impaired in mildly affected muscles, particularly in dystrophinopathies, and may indicate functional muscle ischemia. We aimed to determine if muscle blood flow is reduced in patients with neuromuscular disorders and preserved muscle strength, and if it correlates with echointensity and digital echotexture measurements.

**Methods:**

Peak exercise‐induced blood flow, echointensity, and echotexture were quantified in the elbow flexor muscles of 15 adult patients with Becker muscular dystrophy (BMD) and 13 patients with other muscular dystrophies (OMD). These were compared to 17 patients with Charcot–Marie–Tooth type 1 (CMT1) neuropathy and 21 healthy adults from a previous study.

**Results:**

Muscle blood flow was reduced in all patient groups compared to controls, most prominently in BMD patients (*p* < 0.0001). Echointensity was similarly increased in all patient groups (*p* < 0.05), while echotexture was reduced only in muscular dystrophy patients (p ≤ 0.002). In BMD, blood flow correlated with echotexture (Pearson *r* = 0.6098, *p* = 0.0158) and strength (Spearman *r* = 0.5471; *p* = 0.0370). In patients with normal muscle strength, reduced muscle blood flow was evident in all patient groups (*p* < 0.001), echotexture was reduced in BMD and OMD (*p* < 0.01), and echointensity was increased in CMT (*p* < 0.05).

**Interpretation:**

Muscle blood flow is a sensitive measure to detect abnormality, even in muscles with normal strength. Increased echointensity may indicate a neurogenic disorder when strength is preserved, while low echotexture suggests a dystrophic disease.

## Introduction

Ultrasound is increasingly used in neuromuscular clinics to detect muscle pathology. Neuromuscular disease commonly results in muscle atrophy, fibrosis, and fatty infiltration.^1^ These can be detected by ultrasonography, quantified by measurement of muscle thickness and mean grayscale echointensity, and semiquantified by inspection of changes in muscle texture.^1^, ^2^, ^3^, ^4^ The axial sonographic echotexture of intact muscle is typically a heterogeneous combination of dark muscle fiber tissue and speckles of bright fascia structures, termed “starry night” appearance.^5^ Echotexture changes differently in myopathic and neurogenic disorders, showing a homogeneous increase in echointensity in muscular dystrophy, and nonhomogeneous (i.e., heterogeneous) echointensity in neurogenic disorders.^2^, ^6^, ^7^ Echotexture heterogeneity may be digitally expressed by the bell‐shaped grayscale histogram of an analyzed region of interest,^6^, ^8^ which becomes wider when this is reduced and correspondingly shows a lower standard deviation of echointensity. A low standard deviation of echointensity therefore indicates reduced muscle echotexture.

The utility of muscle ultrasound in the assessment of patients with neuromuscular disorders was most commonly tested in pediatric patients, with a reported sensitivity of 81% and specificity of 96% for visual inspection^9^ and 71–83% sensitivity and 79–91% specificity for quantitative echointensity assessment.^10^, ^11^ However, in a recent comparison to electromyography, both quantified echointensity and visual texture inspection showed low yield, particularly when abnormalities were subtle.^12^

We previously reported that exercise‐induced intramuscular blood flow, particularly at its peak, is reduced in adults with muscular dystrophies^13^ as well as in patients with polyneuropathy due to Charcot–Marie–Tooth (CMT) Type 1 (CMT1),^14^ as quantified by power Doppler ultrasonography. Regulation of skeletal muscle blood flow is highly important as impaired perfusion may result in muscle ischemia and damage while nonspecific perfusion may result with hypotension. Intramuscular blood flow in resting muscles is limited by sympathetic activation of alpha‐adrenergic receptors on local vascular smooth muscle, resulting in vasoconstriction.^15^ Therefore, blood flow to skeletal muscles is minimal at rest, matching a low metabolic demand. Exercise of skeletal muscle releases nitric oxide (NO), which is predominantly produced by sarcolemmal neuronal NO synthase (nNOS) and is key to the attenuation of local sympathetic vasoconstriction, termed functional sympatholysis.^16^, ^17^, ^18^, ^19^ Sarcolemmal nNOS may be reduced in a variety of inherited and acquired myopathic disorders, as well as with neurogenic conditions.^20^

Becker muscular dystrophy (BMD), like the closely related Duchenne muscular dystrophy (DMD), is caused by variants in the dystrophin (*DMD*) gene, resulting in progressive muscle degeneration and muscle weakness.^21^ Most disease causing variants of the X‐linked disease are intragenic deletion or duplications, while the rest are attributable to single nucleotide variants (SNVs). While DMD results in complete loss of dystrophin and severe muscle damage, in BMD the protein is impaired but expressed.^22^

Dystrophin is important for stabilization of the sarcolemma and its deficiency results in fiber damage with repeated contraction.^23^, ^24^ Dystrophin is also required for targeting many proteins to the sarcolemma, including nNOS^25^ which binds spectrin‐like repeats in its rod domain.^26^ Dystrophin deficiency, therefore, causes sarcolemmal nNOS deficiency and functional muscle ischemia.^27^, ^28^, ^29^ In BMD, in‐frame deletions produce a truncated dystrophin protein, which commonly lacks a portion of the rod domain and thus retains some of its functions and shows a milder and variable phenotype.^30^ Accordingly, we previously showed that exercise‐induced muscle blood flow is reduced in DMD and BMD patients and corresponds to disease severity.^31^ However, it remained undetermined if blood flow in muscles is reduced in muscular dystrophy patients with preserved muscle strength.

In the present study, we aimed to determine whether exercise‐induced intramuscular blood flow is reduced in adult patients with muscular dystrophies, and if this can be identified in muscles that produce normal strength. We employed power Doppler to quantify muscle blood flow and grayscale echointensity and echotexture measures to quantify degenerative changes. The study compared patients with BMD, which we expected to show low muscle blood flow, to patients with various other muscular dystrophies (OMD), CMT, and healthy controls. This was done to determine if muscle blood flow, in combination with other structural echointensity variables can differentiate between myopathic and neurogenic disorders.

## Methods

### Participants and study design

This is a cross‐sectional study. We quantified intramuscular blood flow and echointensity in adult patients with BMD and patients with OMD. Data of CMT patients and healthy controls was extracted from a previously published series,^14^ reanalyzed and evaluated in comparison to BMD and OMD patients. In addition to confirmed genetic diagnosis of BMD, all patients were ambulatory at age 13. Controls were defined according to self‐report of no clinical symptoms related to myopathy or other neuromuscular disorder, and intact neurological examination. Participants were recruited in a convenience sampling fashion from December 2020 to October 2022 at the neuromuscular clinic at Sheba Medical Center. The study was approved by the Sheba institutional Helsinki committee and written informed consent was obtained from all participants. The inclusion criterion for the patient groups was documented genetic disease (BMD or other muscular dystrophy). Patients who were limited by an additional disease were excluded.

### Muscle strength testing and exercise protocol

Manual muscle testing (MMT) of the biceps brachii and brachialis strength was graded according to the Medical Research Council (MRC) scoring system by the treating neurologist (A.D.). For the exercise protocol participants semi‐reclined in a supine position with the head of the bed elevated to 30°. The elbow was repeatedly flexed and extended 10 times during 30 seconds. Participants with normal strength performed the exercise while holding a weight, 5 kg for males and 3 kg for females. Participants with an MRC score of 4 held a weight of 3 kg for male and 1 kg for females, and those with a score of 3 or below performed the exercise against gravity or with gravity eliminated, respectively, without holding a weight.

### Ultrasonography protocol

Ultrasound examination was performed by trained physiotherapists (O.G. and T.A.) which were aware of participant's diagnosis but not of clinical details. We employed a Philips Affiniti 70 imaging system with a L18‐5 linear probe as previously described.^14^ Briefly, muscle evaluation was performed while participants were rested in a supine position, arms at their side, with the elbow straight and the forearm supinated. The ultrasound transducer was placed perpendicular to the tested muscle to obtain axial images at the belly portion of the biceps brachii and brachialis muscles. Intramuscular blood flow was quantified similarly to that previously described.^14^ Briefly, power Doppler videos were recorded in 10‐sec intervals. One interval was recorded at rest and six successive intervals were recorded immediately following the exercise protocol. Intramuscular blood flow during the intervals was quantified using the QLAB 10.5 software, employing a microvascular imaging (MVI) tool. The analyzed region of interest included both biceps brachii and brachialis muscles down to the bone line as in patients with muscle degenerative changes discrimination between these muscles was not possible. The brachial artery and vain were excluded. Exercise‐induced intramuscular blood flow was defined as the mean percentage of the area occupied by blood within the muscle during 10 s of peak blood flow.^14^ Within each group, no statistical difference between muscle blood flow in the left and right side was noted, therefore, for each patient an average between the right and left side was calculated. Echointensity quantification of muscle images during rest was performed using ImageJ software (National Institutes of Health; Bethesda, MD) employing a histogram analysis tool. This provided the mean echointensity on a 0–255 gray scale (0 = black, 255 = white) and the standard deviation of the echointensity which was used to quantify the echotexture, and the average between sides was calculated.

### Statistics

Statistical analyses and graphs were conducted using GraphPad Prism (version 9.5.1 for Windows, GraphPad Software, La Jolla, CA, USA). Results are expressed as mean ± SD. For continuous variables, a two‐way ANOVA followed by a post hoc test was applied. Dunnett's multiple comparisons test was used to compare between the two muscular dystrophy groups. Pearson correlation coefficient was used to determine the statistical association between parametric variables and Spearman rank correlation for non‐parametric data. When Bartlett's test was found to be significant the datasets were assessed for normality distribution by the Shapiro–Wilk (W) test. The non‐parametric Kruskal–Wallis test was applied to non‐normal distributed datasets and was followed by the post hoc Dunn's multiple comparisons test.

## Results

### Demographics and genetic data

Patients with BMD (*n* = 15; Table S1) and OMD (*n* = 13; Table S2) were recruited for this study. All BMD patients were males, two were brothers, and all had a confirmed molecular diagnosis (hemizygous) consistent with this condition. Eleven had in‐frame deletion and three had an in‐frame duplication in the DMD gene. We used LOVD exonic deletion/duplication reading frame checker (https://www.dmd.nl) to confirm this. A single patient had the splice site variant c.31+1G>C.

The OMD group included 10 males, none were related. For myotonic dystrophy Type 1 (*n* = 5) and Type 2 (*n* = 2), the pathogenic repeat size was according to the accepted threshold for each disease. For facioscapulohumeral muscular dystrophy (FSHD) type 1 (*n* = 3), the diagnosis was according to Tawil et al. recommendations.^32^ Three others had single nucleotide variants (SNVs) each: c.5G>T, p.Ser2Ile in *DES* (heterozygous), c.4309G>C, p.Ala1437Pro in *MYH7* (heterozygous), and c.4989_4993delinsCCCC, p.Glu1663 AspfsTer10 in *DYSF* (homozygous).

The findings of these patients were compared to data extracted from our previously published series of 21 healthy volunteers (8 males) and 17 patients with CMT1 polyneuropathy (8 males) which employed the same technique, protocol, and ultrasound settings.^14^ Fifteen of the CMT1 patients had duplication of the PMP2 gene, consistent with CMT type 1A. Two had the NM_000530.8:c.397C>T p.Pro133Ser variant in MPZ (heterozygous), consistent with CMT type 1B. All SNVs were classified as pathogenic/likely pathogenic according to the American college of medical genetics and genomics (ACMGG) recommendations.^33^

The mean age of participants was similar between groups, with 31.07 ± 9.98 (range 21–49) in the BMD group, 36.54 ± 11.90 (range 23–66) in the OMD group, 41.71 ± 17.16 (range 19–64) in the CMT group, and 39.52 ± 12.64 (range 21–60) in the controls (*F*(3,62) = 1.914, *p* = 0.1365). We assessed 30 muscles in the BMD group, 26 muscles in the OMD group, 34 muscles in the CMT group, and 42 muscles in the healthy controls.

### Ultrasonography parameters

**Figure 1:**
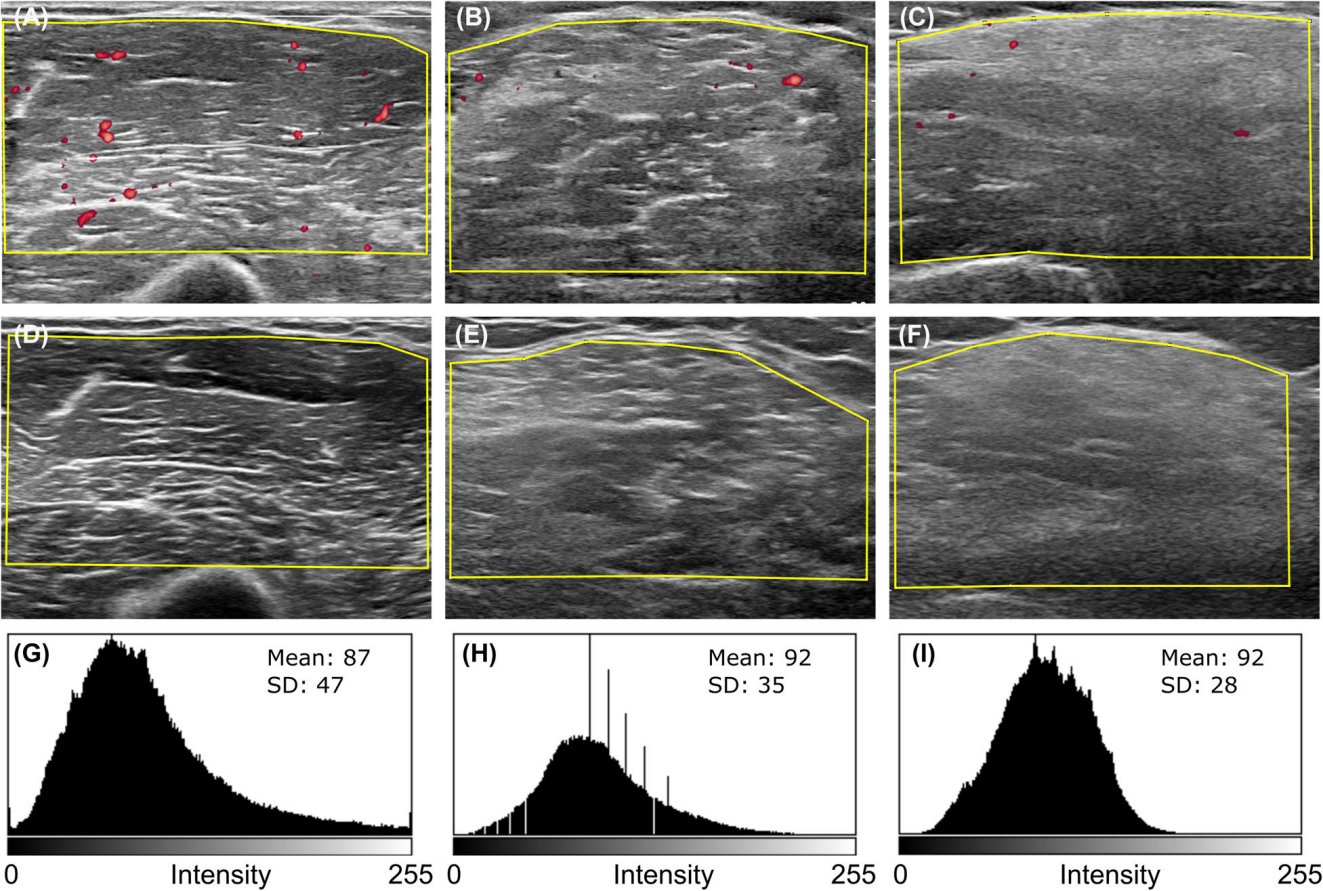
Power Doppler and ultrasound imaging of the elbow flexor muscles. A healthy control muscle (A) shows prominent blood flow, while this is reduced in a BMD patient (B), and in a patient with a dysferlinopathy (C). The echotexture of a normal muscle is heterogeneously speckled (D), attenuated in muscular dystrophy patients (E and F). Histogram analysis of the corresponding above‐placed photos shows the echotexture distribution pattern, the mean echointensity value, and the standard deviation. In contrast with the healthy control (G), both BMD (H) and dysferlinopathy (I) patients show increased mean and reduced standard deviation of echointensity. The X axis of the histograms represents echointensity values between 0 and 255, and the Y axis represents the frequency of observations. BMD, Becker muscular dystrophy; SD, standard deviation.

**Figure 2:**
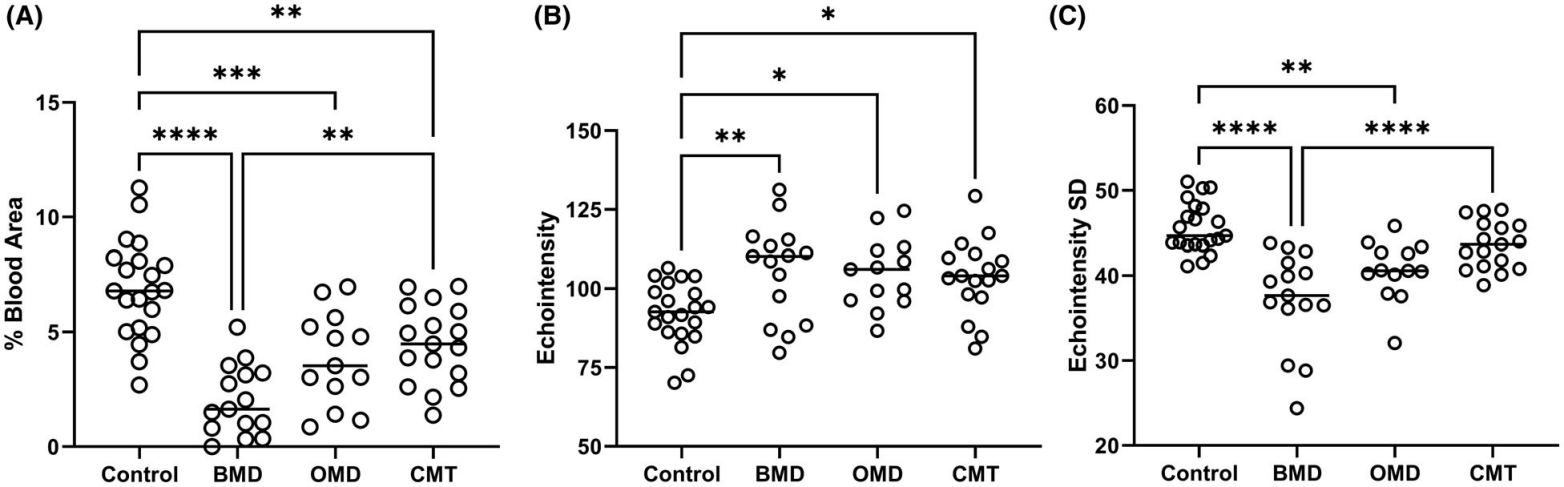
Exercise‐induced intramuscular blood flow, muscle echointensity, and echotexture heterogeneity quantification. Blood flow as % blood in an area of muscle (A), mean echointensity (B), and echotexture as the standard deviation of echointensity histogram analysis (C) are shown. ANOVA Tukey's multiple comparisons ****p < 0.0001; ***p = 0.0001; **p < 0.01; * *p* < 0.05. BMD, Becker muscular dystrophy; CMT, Charcot–Marie–Tooth; OMD, other muscular dystrophy; SD, standard deviation.

Intramuscular blood flow was quantified at rest and after a short exercise protocol. In all groups, intramuscular blood flow at rest was minimal, nearly undetectable. No significant differences were found between the groups. Following exercise muscle blood flow increased in all patient groups (Fig. 1A–C), but was significantly low compared to that of controls (Fig. 2A; Table 1; *F*(3,62) =20.69, *p* < 0.0001, one‐way ANOVA). The reduction of muscle blood flow was most prominent in patients with BMD (BMD vs. controls *p* < 0.0001, BMD vs. OMD *p* = 0.0390, BMD vs. CMT *p* = 0.0015, Dunnett's multiple comparison test).

**Table 1:** Sonographic results.

Group	% Blood area	Echointensity gray scale	Echointensity SD
Controls			
Mean	6.869	92	46
SD	2.140	10	3
95% CI	5.895–7.843	88–97	44–47
BMD			
Mean	2.033	106	37
SD	1.528	15	6
95% CI	1.186–2.879	97–114	34–40
OMD			
Mean	3.827	105	41
SD	2.038	11	3
95% CI	2.596–5.059	98–112	39–43
CMT			
Mean	4.478	104	44
SD	1.720	12	3
95% CI	3.593–5.362	98–110	42–45
BMD with normal strength			
Mean	2.458	104	39
SD	1.527	17	4
95% CI	1.365–3.551	91–116	37–43
OMD with normal strength			
Mean	3.874	104	41
SD	1.662	11	3
95% CI	2.597–5.152	95–113	39–43
CMT with normal strength			
Mean	4.671	103	44
SD	1.574	12	3
95% CI	3.833–5.510	97–110	42–45

BMD, Becker muscular dystrophy; CI, confidence interval of mean; CMT, Charcot–Marie–Tooth; OMD, other muscular dystrophy; SD, standard deviation.

To determine if muscle blood flow reduction correlated with the extent of degenerative changes in muscles, we measured their echointensity (Fig. 1D–F; Table 1). The echointensity was significantly increased in all patient groups compared to healthy controls (*F*(3,62) = 5.164, *p* = 0.0030, one‐way ANOVA) but was similar between BMD, OMD, and CMT patients (Fig. 2B). Nevertheless, muscle texture patterns appeared different between groups, with a more homogeneous pattern in the dystrophies (Fig. 1E,F). Echotexture heterogeneity, determined by the standard deviation of echointensity, was reduced in BMD and OMD patients, in comparison to controls (*p* < 0.0001 and *p* = 0.002, respectively; Fig. 2C) while in CMT patients it was similar to the controls (*p* = 0.3469; Table 1). A significant difference between the BMD and the CMT groups was also found (*p* < 0.0001; Fig. 2C). Multiple comparison analysis to BMD patients indicated more pronounced echotexture changes in this group (BMD vs. OMD *p* = 0.0492; BMD vs. CMT *p* < 0.0001, Dunnett's multiple comparisons test).

**Figure 3:**
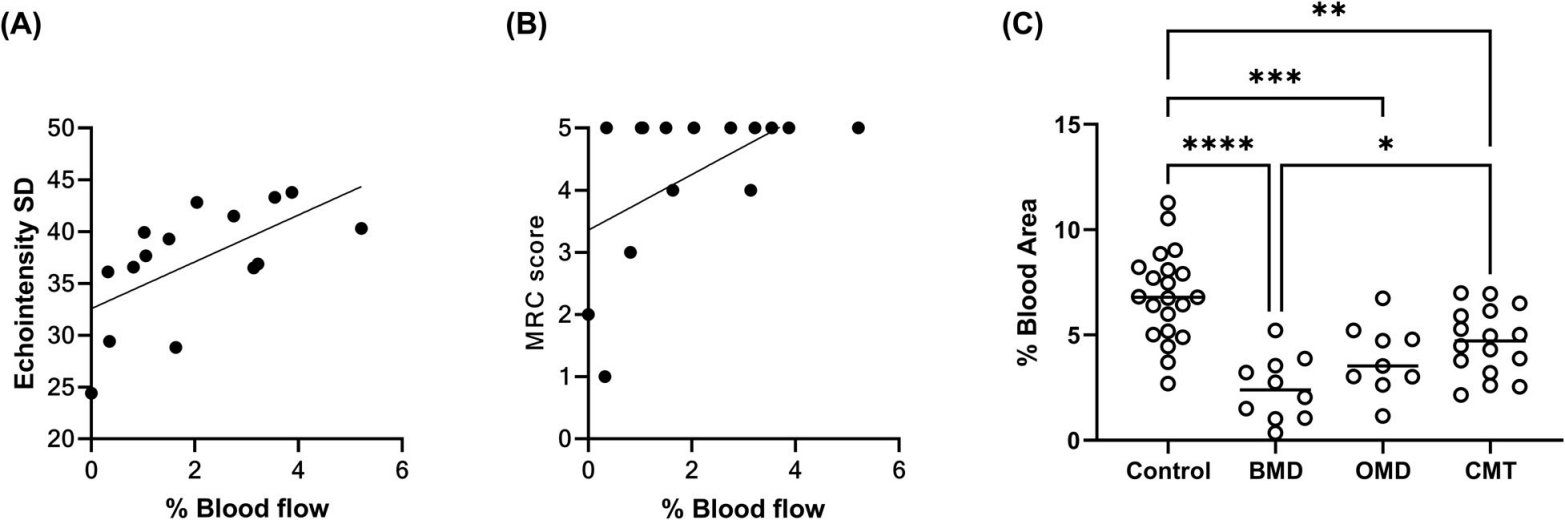
Correlation of intramuscular blood flow with echotexture and muscle strength. In BMD patients blood flow correlated with (A) echotexture heterogeneity (Pearson *r* = 0.6098, *p* = 0.0158, two‐tailed analysis) and with (B) muscle strength (Spearman *r* = 0.5471; *p* = 0.0370, two‐tailed analysis). Blood flow in controls and patients with normal muscle strength (C). ****p < 0.0001; ***p = 0.0007; **p < 0.01; *p < 0.05. BMD, Becker muscular dystrophy; CMT, Charcot–Marie–Tooth; OMD, other muscular dystrophy; SD, standard deviation.

To determine whether the reduction of muscle blood flow is associated with muscle degenerative changes, we performed correlation tests between the muscle blood flow and echointensity parameters. No significant correlation was found between muscle blood flow and echointensity in all the four groups (Table S3). A significant correlation was found between muscle blood flow and echotexture in the muscles of BMD patients (Fig. 3A, Table S3). This suggested that in BMD patients, the uniform degenerative changes correlate with the limitation of blood flow into the muscle. To compare muscle blood flow in patients with similar extent of degenerative changes we excluded three BMD patients with an extremely low echotexture heterogeneity (echointensity SD below 30). This showed that muscle blood flow was similarly reduced in BMD and OMD patients with mild degenerative changes (BMD vs. OMD *p* = 0.1426, Dunnett's multiple comparisons).

To evaluate if muscle blood flow correlated with muscle strength we tested its association with the MMT score. Patients with BMD showed a moderate correlation between muscle blood flow and strength (r = 0.5471, *p* = 0.0370, Fig. 3B). Most BMD patients showed normal elbow flexion strength, yet with limited muscle blood flow (Table S4). A comparison of patients with normal elbow flexion strength (10 BMD, 9 OMD, 16 CMT, and 21 controls) showed that muscle blood flow was reduced in all patient groups (Fig. 3C; BMD *p* < 0.0001, OMD *p* = 0.0007, CMT *p* = 0.0034), and was similar in BMD and OMD patients (BMD vs. OMD *p* = 0.2140 Dunnett's multiple comparisons; Table 1). In patients with normal strength, the echotexture was reduced in BMD (*p* < 0.0001) and OMD (*p* = 0.0026) groups but not in CMT, and echointensity was increased only in CMT (*p* = 0.0439).

No correlation was found between muscle blood flow in the elbow flexors and age in all the four groups (Table S3). To rule out an effect of sex on the analysis results, we repeated it among males only (10 BMD, 5 OMD, 8 CMT, and 8 controls). This underpowered analysis showed similar results, indicating low muscle blood flow and echotexture predominantly in BMD patients (Fig. S1).

**Figure 4:**
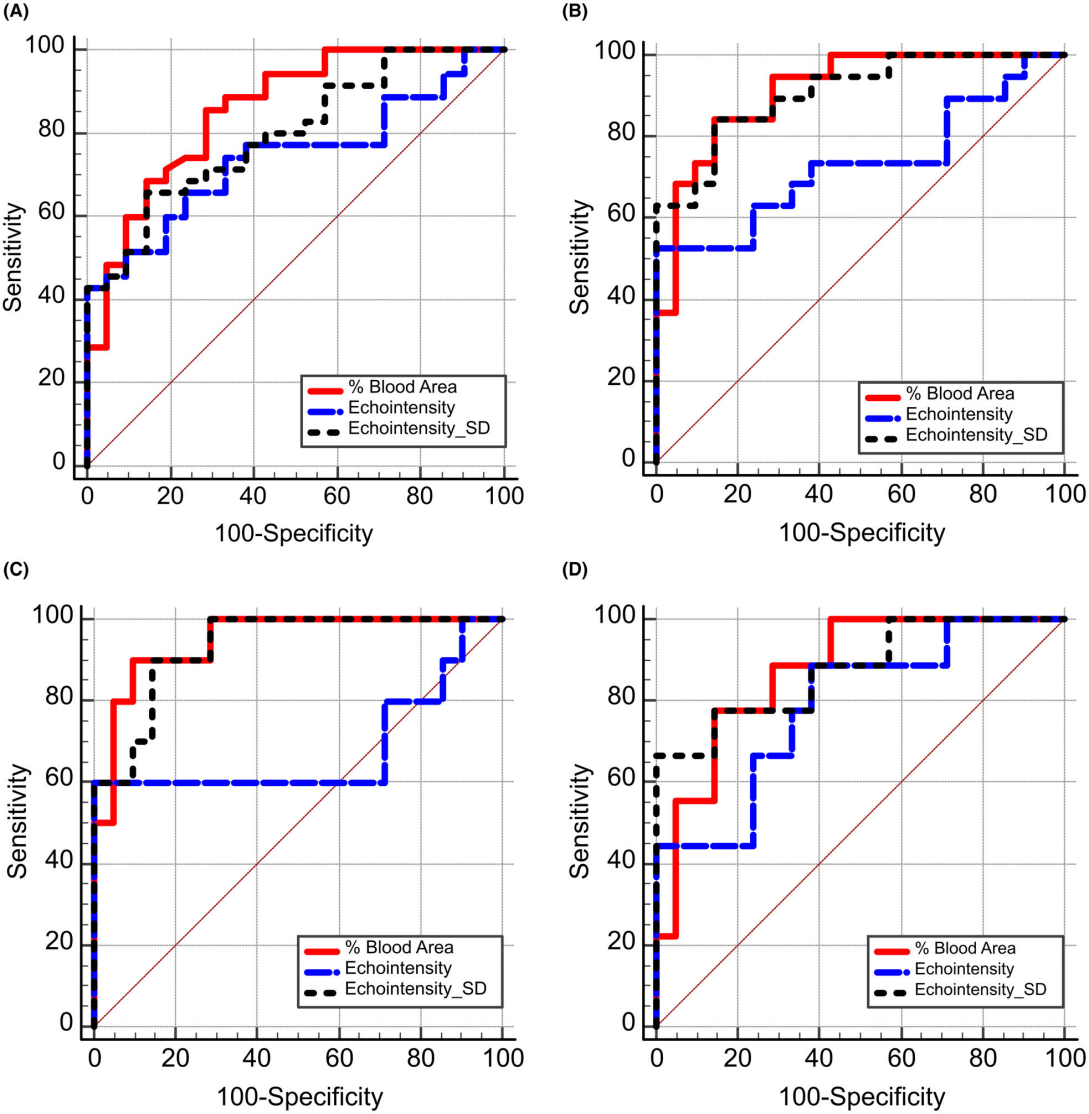
Receiver operator characteristics. Blood flow (% blood area) evaluation showed a higher area under the curve (AUC) for detecting an abnormality in comparison to echointensity and echotexture (echointensity SD) analysis in our entire mixed patient population with normal strength (A), muscular dystrophy patients with normal elbow flexion strength (B), BMD patients with normal strength (C), and patients with OMD with normal strength (D). BMD, Becker muscular dystrophy; OMD, other muscular dystrophy; SD, standard deviation.

We evaluated the sensitivity and specificity of muscle blood flow, echointensity, and echotexture measurements to detect a neuromuscular disorder with receiver operator characteristics (ROC) analysis (Table S4). Muscle blood flow evaluation showed a higher area under the curve (AUC) for detection of abnormality in comparison to echointensity and echotexture analysis in our whole mixed patient sample with normal strength (Fig. 4A), in muscular dystrophy patients with normal elbow flexion strength (Fig. 4B) and particularly in BMD patients with normal strength (Fig. 4C). In patients with OMD and normal strength the AUC was similar for muscle blood flow and echotexture measurements (Fig. 4D). In all scenarios, measurements of muscle blood flow and echotexture were superior to echointensity for detection of an abnormality.

## Discussion

Muscle ultrasound is a noninvasive bedside assessment tool for detection and initial characterization of a neuromuscular disease. High sensitivity of this tool is particularly important when muscle weakness and atrophy are not clinically evident. However, the commonly used parameters of increased mean echointensity and semiquantitative echotexture changes are not sensitive to detect mild muscle pathology, as these relate to chronic structural changes. In this study, we employed a physiologic parameter to detect muscle dysfunction. We showed that compared to controls, exercise‐induced muscle blood flow is reduced in patients with muscular dystrophies, particularly in BMD, and that reduction of muscle blood flow is a sensitive measure, identified in muscles with normal strength and only mild degenerative changes. Furthermore, while increased mean echointensity is nonspecific, we showed that attenuation of texture may be quantified by the standard deviation of echointensity, and that this is more sensitive and specific than echointensity for detecting a muscular dystrophy. While the changes in echointensity and texture are expected from previous studies, the evidence for early detection of muscle disease with power Doppler is novel.

Skeletal muscle blood flow is tightly regulated, as impaired perfusion may result in muscle ischemia and damage.^34^ Interestingly, muscle blood flow restriction can elicit muscle hypertrophy when combined with low‐load exercise (30% maximal voluntary contraction) training.^35^ Skeletal muscle hypertrophy in response to resistance exercise employs multiple factors.^36^ One of them is NO, which in addition to its role in exercise‐induced sympatholysis, promotes activation of satellite cells, and is implicated in aging and sarcopenia in animal models.^37^, ^38^, ^39^

Functional muscle ischemia due to dysregulated blood flow to the muscle is a prominent feature in dystrophinopathies due to the absence of dystrophin in DMD and variable reduction in BMD. This results in the absence or reduction of sarcolemmal nNOS and reduced excretion of NO^40^. NO acts through the second messenger cyclic guanosine monophosphate (cGMP) and levels of cGMP are regulated through degradation, mainly by phosphodiesterase type 5 (PDE5). Multiple preclinical animal model studies showed that inhibition of cGMP degradation by PDE5 inhibitors was beneficial to skeletal, respiratory, and cardiac muscles, improved endurance training performance, and enhanced microvascular function.^40^, ^41^, ^42^, ^43^ These findings suggested PDE5‐inhibitors to be a putative new treatment for DMD. However, controlled clinical studies with PDE5 inhibitors failed to show improvements in clinical outcomes, in both DMD and BMD patients.^44^, ^45^, ^46^ The disappointing lack of effect was assumed to be due to low levels of the target PDE5 protein in dystrophic muscle.^44^ Our study provides physiological evidence that functional ischemia occurs in BMD as well as in patients with OMD, as well as patients with neuropathy. Identifying functional muscle ischemia in muscles with normal strength and mild degenerative changes implies that this therapeutic approach, based on PDE5 inhibitors, may be suitable for patients with normal or near‐normal strength, and mild degenerative changes on imaging studies. Furthermore, in future studies, muscle blood flow quantification with power Doppler may be employed to identify a pharmacological effect in humans, in a simple bedside manner. Nevertheless, as functional muscle ischemia in patients with muscular dystrophy is not the sole cause of pathology,^47^ its potential efficacy is probably limited, and should not be expected to completely resolve contraction‐induced myofiber damage.

Our study has several limitations. One is the small patient groups, which are further reduced to study patients with muscular dystrophy and normal muscle strength. Nevertheless, comparisons to healthy controls do show statistically validated results, despite the small number of participants. There was no statistical significance between groups in mean age, and thus we did not control this variable in the analysis. More importantly, as the BMD group included only males, while the other three patient groups included also females, we performed a secondary analysis that excluded females. In this male only sub analysis, the results were similar to the results of the primary analysis, though with limited statistical significance. Second, power Doppler measurements are readily affected by the extent of muscle compression by the ultrasound probe. This may give rise to a large variation when examining “soft” muscles, commonly due to denervation, but may also affect “hard” muscles with extensive fibrosis. We did not use any pressure measurement device to unify this variable, but rather maintained a high level of awareness of this possible artifact, and maintained the lowest possible applied pressure to obtain images with full skin contact, which was shown to provide low bias for muscle volume measurements.^48^ Third, we did not standardize the resistance and repeats of the exercise according to the strength of one maximal repetition (1RM). This was avoided in order to prevent possible muscle damage in dystrophy patients due to exertion of maximal strength. We employed the MRC score documented in the corresponding clinical visit to determine the strength and the applied exercise weight, but this score is not a sensitive test of normal muscle strength. Thus, muscles with mild echointensity and/or echotexture abnormality may perhaps show weakness on more standardized exercise measurements. Last, we measured echointensity and power Doppler signals in a region of interest that extended from the superficial surface of the biceps brachii muscle down to the humerus bone line, including portions of both the biceps brachii and brachialis muscles. As ultrasound signals attenuate with increasing depth, particularly in pathologic muscle, the deeper portions of the region of interest would not accurately reflect muscle texture. This approach was adopted to uniformly assess the elbow flexor muscles even when distinction between them is not possible. Perhaps in cohorts that are focused on patients with normal muscle strength, analysis of only the biceps brachii or its superficial portion may be more accurate.

In conclusion, the findings of our study highlight that exercise‐induced muscle blood flow is a sensitive measure to detect a neuromuscular abnormality. Therefore, adding power Doppler assessment to echointensity measurements may increase the yield of ultrasonographic evaluation of a neuromuscular disorder, particularly at an initial phase of pathology, when muscle weakness and atrophy are not clinically identified. Together with echointensity and echotexture measurements, muscle blood flow may serve as a quantifiable sensitive diagnostic biomarker of functional muscle ischemia and muscle degeneration.

## Author Contributions

Amir Dori and Orna Gera: Conception, design, data analysis, and editing. Efrat Shavit‐Stein and Lior Greenbaum: Design, data analysis, and editing. Taly Amichai and Odelia Chorin: Design and data analysis. Joab Chapman: Conception and editing.

## Funding Information

No funding was received for this study.

## Conflict of Interest

None of the authors have any conflict of interest to disclose.

## Supporting information


Appendix S1.

## Data Availability

The data that support the findings of this study are available from the corresponding author upon reasonable request.
