# Supplementary material for: Muscular dystrophy patients show low exercise‐induced blood flow in muscles with normal strength
Source: Ann Clin Transl Neurol. 2024 Sep 9;11(11):2866–76. doi: 10.1002/acn3.52194 (PMC11572729; doi:10.1002/acn3.52194)
Supplement: Supplementary file 1 — Appendix S1. [file ACN3-11-2866-s001.docx]

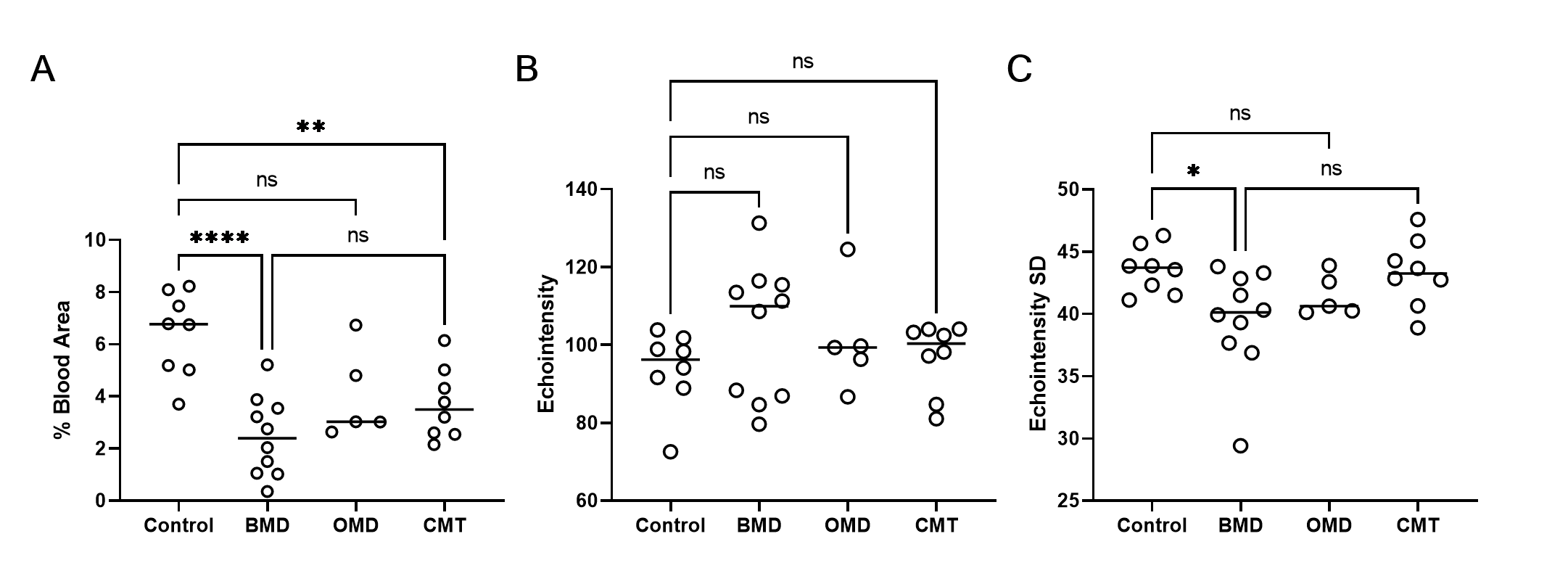


**Supplementary Figure 1.** Analysis of male subjects with normal muscle strength. Muscle blood flow (A) was low in BMD and CMT patients versus controls, and marginally low in OMD patients (p=0.0565). Echointensity (B) was similar in patient groups to that of controls. Echotexture (C) was reduced in BMD versus controls. ****p<0.0001; **p<0.01; * p<0.05.

Abbreviations: BMD, Becker muscular dystrophy; CMT, Charcot–Marie–Tooth; OMD, other muscular dystrophy; SD, standard deviation.

**Supplementary Tables**

| Case | Age | *DMD* variant (hemizygous) | Ambulation | MRC |
| --- | --- | --- | --- | --- |
| 1 | 25 | Exons 45-55, in-frame deletion | With crutches | 5 |
| 2 | 33 | Exons 45-46 in-frame deletion | No | 1 |
| 3 | 26 | Exons 45-46 in-frame deletion | No | 2 |
| 4 | 38 | Exons 45-47 in-frame deletion | With crutches | 5 |
| 5 | 49 | Exons 45-47 in-frame deletion | With crutches | 5 |
| 6 | 24 | Exons 45-48 in-frame deletion | Yes | 5 |
| 7 | 49 | Exons 45-48 in-frame deletion | Yes | 5 |
| 8 | 24 | Exons 45-49 in-frame deletion | No | 4 |
| 9 | 21 | Exons 45-51 in-frame deletion | Yes | 5 |
| 10 | 26 | Exons 45-51 in-frame deletion | Yes | 5 |
| 11 | 37 | Exons 45-51 in-frame deletion | Yes | 5 |
| 12 | 23 | Exons 2-7 in-frame duplication | Yes | 4 |
| 13 | 23 | Exons 2-7 in-frame duplication | Yes | 5 |
| 14 | 46 | Exon 3 in-frame duplication | With support | 3 |
| 15 | 27 | NM_004006.3: c.31+1 G>C | Yes | 5 |

**Supplementary Table 1.** Becker muscular dystrophy patients’ characteristics. DMD, Duchenne muscular dystrophy gene.

MRC, Medical Research Council score.

| Case | Age | Gender | Myopathy type | MRC |
| --- | --- | --- | --- | --- |
| 1 | 28 | Male | DM1 | 5 |
| 2 | 35 | Male | DM1 | 5 |
| 3 | 42 | Female | DM1 | 5 |
| 5 | 23 | Male | DM1 | 5 |
| 7 | 36 | Male | DM1 | 5 |
| 4 | 36 | Female | DM2 | 5 |
| 6 | 49 | Male | DM2 | 5 |
| 8 | 23 | Male | FSHD1 | 5 |
| 9 | 66 | Male | FSHD1 | 5 |
| 10 | 35 | Male | FSHD1 | 4 |
| 11 | 43 | Female | Myofibrillar myopathy (het. NM_001927.4:c.5G>T, p.Ser2Ile variant in *DES*) | 5 |
| 12 | 24 | Male | LGMD R2 (hom. NM_001130987.2: c.4989_4993delinsCCCC, p.Glu1663AspfsTe10 variant in *DYSF*) | 4 |
| 13 | 35 | Male | MYH7-related myopathy/dystrophy (het. NM_000257.4: c.4309G>C, p.Ala1437Pro variant in *MYH7*) | 5 |

**Supplementary Table 2.** Other muscular dystrophy patients’ characteristics.

FSHD, facio-scapulo-humeral muscular dystrophy; LGMD, limb-girdle muscular dystrophy; DM, myotonic dystrophy; MRC, Medical Research Council score; MYH, Myosin Heavy Chain.

|  |  | Pearson correlation r | | | | Pearson correlation p | | | |
| --- | --- | --- | --- | --- | --- | --- | --- | --- | --- |
| Group |  | % Blood Area | Echo-intensity Gray | Echo-intensity SD | Age | % Blood Area | Echo-intensity Gray | Echo-intensity SD | Age |
| Controls | % Blood Area | 1.000 | -0.102 | 0.391 | -0.008 |  | 0.659 | 0.080 | 0.972 |
|  | Echo-intensity | -0.102 | 1.000 | 0.018 | 0.181 | 0.659 |  | 0.938 | 0.431 |
|  | Echo-intensity SD | 0.391 | 0.018 | 1.000 | -0.090 | 0.080 | 0.938 |  | 0.698 |
|  | Age | -0.008 | 0.181 | -0.090 | 1.000 | 0.972 | 0.431 | 0.698 |  |
| BMD | % Blood Area | 1.000 | -0.043 | 0.610 | -0.157 |  | 0.880 | **0.016** | 0.576 |
|  | Echo-intensity | -0.043 | 1.000 | -0.591 | 0.328 | 0.880 |  | **0.020** | 0.232 |
|  | Echo-intensity SD | 0.610 | -0.591 | 1.000 | -0.142 | **0.016** | **0.020** |  | 0.614 |
|  | Age | -0.157 | 0.328 | -0.142 | 1.000 | 0.576 | 0.232 | 0.614 |  |
| OMD | % Blood Area | 1.000 | 0.147 | 0.360 | 0.394 |  | 0.633 | 0.227 | 0.183 |
|  | Echo-intensity | 0.147 | 1.000 | 0.087 | 0.349 | 0.633 |  | 0.778 | 0.243 |
|  | Echo-intensity SD | 0.360 | 0.087 | 1.000 | 0.250 | 0.227 | 0.778 |  | 0.410 |
|  | Age | 0.394 | 0.349 | 0.250 | 1.000 | 0.183 | 0.243 | 0.410 |  |
| CMT | % Blood Area | 1.000 | 0.292 | 0.331 | 0.050 |  | 0.255 | 0.195 | 0.849 |
|  | Echo-intensity | 0.292 | 1.000 | 0.394 | 0.492 | 0.255 |  | 0.118 | **0.045** |
|  | Echo-intensity SD | 0.331 | 0.394 | 1.000 | -0.019 | 0.195 | 0.118 |  | 0.943 |
|  | Age | 0.050 | 0.492 | -0.019 | 1.000 | 0.849 | **0.045** | 0.943 |  |

**Supplementary Table 3.** Correlation analysis. P<0.05 is marked in bold.

Abbreviations: BMD, Becker muscular dystrophy; CMT, Charcot–Marie–Tooth; OMD, other muscular dystrophy; SD, standard deviation of echointensity.

| Case | MRC | % Blood flow | Echointensity gray scale | Echointensity SD |
| --- | --- | --- | --- | --- |
|  |  |  |  |  |
| 1 | 5 | 2.040 | 80 | 43 |
| 2 | 1 | 0.325 | 104 | 36 |
| 3 | 2 | 0.000 | 110 | 24 |
| 4 | 5 | 0.355 | 131 | 29 |
| 5 | 5 | 1.055 | 114 | 38 |
| 6 | 5 | 2.750 | 116 | 42 |
| 7 | 5 | 3.220 | 116 | 37 |
| 8 | 4 | 1.635 | 126 | 29 |
| 9 | 5 | 3.875 | 87 | 44 |
| 10 | 5 | 1.500 | 88 | 39 |
| 11 | 5 | 5.215 | 111 | 40 |
| 12 | 4 | 3.135 | 110 | 37 |
| 13 | 5 | 1.025 | 85 | 40 |
| 14 | 3 | 0.815 | 98 | 37 |
| 15 | 5 | 3.545 | 109 | 43 |

**Supplementary Table 4.** Becker muscular dystrophy patients’ muscle strength, exercise-induced intramuscular blood-flow, echointensity and echotexture heterogeneity.

MRC, Medical Research Council score; SD, standard deviation.

| Group | Variable | AUC | 95% CI | P value | Criterion | Sensitivity | Specificity |
| --- | --- | --- | --- | --- | --- | --- | --- |
| All patients | % Blood Area | 0.868 | 0.778 to 0.958 | <0.0001 | ≤5.91 | 86.67 | 71.43 |
|  | Echointensity | 0.778 | 0.668 to 0.888 | <0.0001 | >104.18 | 53.33 | 95.24 |
|  | Echointensity SD | 0.837 | 0.742 to 0.932 | <0.0001 | ≤43.38 | 73.33 | 85.71 |
| Neuromuscular patients with normal elbow flexion strength | % Blood Area | 0.856 | 0.755 to 0.958 | <0.0001 | ≤5.91 | 85.71 | 71.43 |
|  | Echointensity | 0.741 | 0.613 to 0.870 | =0.0002 | >106.59 | 42.86 | 100.00 |
|  | Echointensity SD | 0.799 | 0.684 to 0.913 | <0.0001 | ≤43.30 | 65.71 | 85.71 |
| BMD with normal elbow flexion strength | % Blood Area | 0.948 | 0.874 to 1.000 | <0.0001 | ≤3.88 | 90 | 90.48 |
|  | Echointensity | 0.681 | 0.416 to 0.946 | =0.1801 | >106.59 | 60 | 100.00 |
|  | Echointensity SD | 0.933 | 0.850 to 1.000 | <0.0001 | ≤43.30 | 90 | 85.71 |
| OMD with normal elbow flexion strength | % Blood Area | 0.873 | 0.744 to 1.000 | <0.0001 | ≤4.80 | 77.78 | 85.71 |
|  | Echointensity | 0.788 | 0.603 to 0.973 | =0.0022 | >94.19 | 88.89 | 61.90 |
|  | Echointensity SD | 0.878 | 0.730 to 1.000 | <0.0001 | ≤40.64 | 66.67 | 100.00 |

**Supplementary Table 5.** Receiver operator characteristics analysis.

Abbreviations: AUC, area under the cure; BMD, Becker muscular dystrophy; CI, confidence interval; CMT, Charcot–Marie–Tooth; OMD, other muscular dystrophy; SD, standard deviation.
